# CCR5Δ32 Polymorphism Associated with a Slower Rate Disease Progression in a Cohort of RR-MS Sicilian Patients

**DOI:** 10.1155/2011/153282

**Published:** 2011-06-23

**Authors:** Rosalia D'Angelo, Concetta Crisafulli, Carmela Rinaldi, Alessia Ruggeri, Aldo Amato, Antonina Sidoti

**Affiliations:** Department of Biomorphology and Biotechnologies, University of Messina, 98125 Messina, Italy

## Abstract

Multiple sclerosis (MS) disease is carried through inflammatory and degenerative stages. Based on clinical feaures, it can be subdivided into three groups: relapsing-remitting MS, secondary progressive MS, and primary progressive MS. Multiple sclerosis has a multifactorial etiology with an interplay of genetic predisposition, environmental factors, and autoimmune inflammatory mechanism in which play a key role CC-chemokines and its receptors. In this paper, we studied the frequency of CCR5 gene Δ32 allele in a cohort of Sicilian RR-MS patients comparing with general Sicilian population. Also, we evaluate the association between this commonly polymorphism and disability development and age of disease onset in the same cohort. Our results show that presence of CCR5Δ32 is significantly associated with expanded disability status scale score (EDSS) but not with age of disease onset.

## 1. Introduction

Multiple sclerosis (MS) is a autoimmune chronic disorder of the central nervous system (CNS) characterized by multifocal inflammatory destruction of myelin, axonal damage, loss of oligodendrocytes, and repair mechanism [1]. 

MS is a frightening and potentially disabling disease for young adults that afflicts around 2.5 million people worldwide with an incidence of about 7 per 100,000 persons every year, a prevalence of around 120 per 100,000, and a lifetime risk of 1 in 400 [2]. 

Studies of MS-twin pairs have revealed that the estimated concordance for MS was 3%–5% for dizygotic and 25%–30% for monozygotic twins [3, 4].

On the basis of the temporal course of disease, MS can be subdivided into three clinical groups: relapsing-remitting MS (RR-MS, characterized by relapses when there is a flare up of symptoms, followed by remissions when there are not symptoms), secondary progressive MS (SP-MS, a slow and gradual form that begins with a relapsing-remitting course), and primary progressive MS (PP-MS, which is expressed, from the start, by a gradual progression of disability). The most common form of disease in approximately 85% of the cases is RR-MS and, typically, the illness passes through phases of relapse with full recovery, relapse with persistent deficit, and secondary progression [1, 2]. Patients with RR-MS accumulate disability from disease onset more slowly than those with primary progressive multiple sclerosis. Most therapeutic agents used in MS (e.g., immunosuppressive and immunomodulatory drugs and cell-cycle interruption drugs) are only used for RR-MS. These treatments show some efficiency in lessening the relapse rate in RR-MS and time to progression but cannot cure MS. Thus, there is a need for new efficient treatments for all types of MS [5]. The etiology of MS is unknown but likely multifactorial, with an interplay of genetic predisposition, environmental factors and autoimmune inflammatory mechanism; in fact, this disease is carried through two stages: inflammatory and degenerative [6, 7]. The migration of inflammatory cells is facilitated by the upregulation of adhesion molecules-1 (ICAM-1), vascular-cell adhesion molecules-1 (VCAM-1), and platelet endothelial cell adhesion molecule-1 (PECAM-1) [8]. On the other hand, matrix metalloproteinases (MMPs) may degrade components of the basement membrane, and chemokines can form concentration gradient, which attract leukocytes and activate leukocyte integrins, increasing adherence and extravasation [9]. Then, the proinflammatory cytokines (e.g., TNF-a, INF-g, IL-2, and IL-12) released by Th 1 cells and macrophages trigger a chain of events, resulting in the formation of demyelinated plaque and damage to axon [8, 10, 11]. Activated T cells enter the CNS and trigger an inflammatory cascade that leads to recruitment of other immune cells. Increasing lines of evidence have implicated an involvement for chemokines and their receptors in several neurodegenerative disorders, including Alzheimer's disease (AD), Parkinson's disease (PD), HIV-associated dementia (HAD), and also in multiple sclerosis (MS) [12]. Genetic studies indicate that CC-chemokines and chemokine receptors are involved in the susceptibility to MS [13], in fact; they play a significant role in the migration of monocytes and T cells. In particular, CC-chemokine receptor 5 (CCR5), a seven-transmembrane spanning G protein-coupled receptor, is a specific binding site for the CC-chemokines like RANTES (CCL5), macrophage inflammatory protein MIP 1*α* (CCL3), and macrophage inflammatory protein MIP 1*β* (CCL4) [14]. CCR5 encoded by CCR5 gene, located on chromosome 3p21 [15], has been identified as a coreceptor for the human immunodeficiency virus-1 (HIV-1) [16]. A nonfunctional allele, resulting from a 32-bp deletion in exon 4, CCR5Δ32, leads to a truncated form of the functional receptor. Individuals homozygous for a 32-bp deletion allele of CCR5, CCR5Δ32, were resistant to HIV-1 infection despite repeated exposure [14]. Moreover, in the CNS, CCR5 is expressed on neurons, astrocytes, and microglia [17], as well as on endothelium and vascular smooth muscle cells and on T-helper cells [18]. During MS pathogenesis, the absence of functional CCR5 on the cell surface could lead to reduced trafficking of leucocytes into the lesion sites, thus downregulating inflammation in brain tissue. To assess the possible association between CCR5Δ32 and the course of MS, various studies were performed, and while several studies showed an association between CCR5Δ32 and a favorable clinical course of MS [19–21], no correlation was found between this polymorphism and the course of the disease in other studies [22–24]; however, the results are in contrast and are not yet clear [25]. Based upon the key role played by chemokines in the migration of macrophages and T cells in MS, and the importance of genetic factors associated and the possible viral or nonviral stimulation involved in the pathogenesis of the disease, in this work, we investigated the effect of CCR5Δ32 on age of onset, course, and severity of disease in cohort of Sicilian patients with RR-MS.

## 2. Materials and Methods

### 2.1. Patient Samples

Peripheral blood specimens were obtained from 180 RR-MS patients (131 female and 49 male) recruited from the Department of Neurosciences, Psychiatric and Anaesthesiological Sciences, University of Messina, Italy. All patients in this study fulfilled the criteria for clinically defined MS [26] and underwent routine diagnostic CSF and blood tests, and a full neurological examination. At the time of sample collection, disease type was defined as relapsing-remitting MS (RR-MS) disease, with Kurtzke expanded disability status scale scores between 0.0 and 6.5 (EDSS-expanded disability status scale or disease progression index). 

Control group comprised 213 unrelated, healthy donors without a family history of autoimmune diseases and were collected during field studies conducted in 1999 and 2005. The samples were taken from several towns in Sicily.

The individual donors were eligible for the present study if their ancestors were born in the same location and if they had no progenitors in common up to their grandparental generation. 

All parameters (age, age at onset, and EDSS) of male versus female were matched in the MS patient group as well as the age of male versus female in the controls. All participants gave their written informed consent.

### 2.2. Genotyping

Genomic DNA was isolated using the salting out method [27]. 

Eparinized peripheral blood was collected from cases and controls before any therapy. PCR was carried out in a thermal cycler (Gene Amp PCR System 9600; PE Applied Biosystems, Foster City, CA) in 50 *μ*L volumes containing a 0.2 *μ*m concentration of each primer (forward CCR5 primer: 5′-GTCTTCATTACACCTGCAGCTC; reverse: 5′-GTGAAGATAAGCCTCACAGCC), 1U Euro Taq polymerase (Euroclone Spa Life Sciences Division, Italy) and 0.8 *μ*g genomic DNA as template, under the following conditions: denaturation at 95°C for 1.5 min, annealing at 56.5°C for 1 min and extension at 72°C for 50 s for 35 cycles, after an initial 5 min denaturation at 95°C. Aliquot of the PCR products were separated using agarose gel electrophoresis, and the 198 bp in the case of the wild-type allele and 166 bp in the case of the Δ32 allele were visualized using ultraviolet light, following ethidium bromide staining.

### 2.3. Statistical Analysis

For each group (control and patients), the CCR5Δ32 allele frequencies were calculated by direct gene counting. Statistical analyses were performed using “Statistica” package [28]. Data are presented as mean ± SD for parametric variables and as percentages for non-parametric values. Continuous variables were compared using Student's *t*-test to test possible influence of CCR5Δ32 allele on EDSS score and age onset. With the aim of reducing possible sources of variance, we included in our analyses gender, age, and age at disease onset as covariates using ANOVA method for statistical analysis. All *P* values were 2-tailed, and statistical significance was set at *P* < .05.

## 3. Results

The demographic data are reported in Table 1 which indicates no significant differences (*P* < .05) between overall MS age versus overall healthy control age. The frequencies of CCR5Δ32 alleles in patients with MS (*fr* = 0.06) did not differ significantly from those of controls (*fr* = 0.06) and were not influenced by gender (*χ* ^2^ = 2.00, *P* = .13). We observed a significant association between CCR5Δ32 allele and EDSS scores (*t* = 2.04, *P* < .05). As a consequence, for reducing possible sources of variance, we introduced all such variables as covariates in our model. Data showed that introducing sex, age, and age of onset as covariates, results remain statistically significant. We did not observed any significant association between CCR5Δ32 allele and age onset (*P* > .05) (Table 2).

## 4. Discussion

Chemokine receptors and their ligands play a key role in several neurodegenerative diseases, such as MS, when they are activated inappropriately [34]. The chemokine receptors expressed by T helper cells may be important reports allowing the association between multiple sclerosis and CNS infiltrated CD4 cells and monocytes. For this reason, it seems that these receptors may be related to the development of new lesions in multiple sclerosis [35].

In addition in a T-cell-mediated inflammatory disease like MS, the expression of chemokines and their receptors is markedly regulated by proinflammatory cytokines, for example, tumour necrosis factors or interleukins [36]. All these evidences are also confirmed by various studies that showing an elevated expression of CC-chemokine in the CNS in MS patients: researchers have focused on chemokine receptor, their ligands, and they have highlighted the role they have played in the development and clinical course of MS [37–39]. In particular, the role of CCR5 in MS is further strengthened by recent finding of elevated CCR5 levels in CD8(+) cells in MS patients [36]. 

From our study, it was found that the CCR5Δ32 allele frequency in Sicilian RR-MS patients (*fr* = 0.06) is comparable to that found in the general population (*fr* = 0.06) [40]. Our results, on the one hand, are consistent with those of some studies [20] and, on the other hand, are not confirmed by other studies [29, 31].

In fact, Otaegui et al. reported a lower frequency of the CCR5Δ32 allele in MS patients compared with the control population in Basque, Spain [31]. Indeed, in the Iranian population, Shahbazi et al. demonstrated that the Δ32 allele frequency was higher among MS patients than in control [29]. These conflicting data may result from several factors as sample size, environmental factors, and differences in ethnicity [41].

With regard to the association Δ32CCR5 allele and clinical course of MS the data in the literature are conflicting. In fact, various studies described an association of the Δ32CCR5 allele with a favorable clinical course of MS [19–21], while others have failed to show any significant correlation between this polymorphism and the course of the disease [22, 23] (an overview of main association studies is summarized in Table 3). Our data showed an association of the CCR5Δ32 allele with expanded disability status scale score (*t* = 2.04, *P* < .05) in RR-MS patients, suggesting that its presence contributes to a slower rate of disease progression. From these results appears a lower expression of a functional CCR5 receptor that might influence the rate of disability development, and then harboring the mutated CCR5 allele can be considered a favorable prognostic factor in MS.

However, as opposed to another recent report [19], we did not find a difference in the age at disease onset between the Δ32CCR5 carriers and noncarriers (*P* > .05). In our opinion, it is important to note that the data shown in this work are not influenced by factors such as age, age of onset disease, and gender (see at the results in Table 2).

Overall, the genetic susceptibility to MS remains an open question. Much of the future research interest will focus to better understand the genetic heterogeneity of this disease and the therapeutic needs individual though the replication of candidate gene association analysis is difficult in MS as is often happens with complex genetic diseases. The broad system of chemokines and their receptors may be an important key in the search for answers to these questions. More studies are needed to reveal the exact role of chemokine network and its association with other factors in the pathogenesis of this disease. Our data suggest that the presence of this CCR5 polymorphism in patients with RR-MS might help in designing a therapeutic regimen and might serve as a prognostic marker and then that CCR5 antagonists could be considered potential targets for therapeutic intervention in MS.

## Figures and Tables

**Table 1:** Demographic and disease-related data of our samples.

	Gender	*n*	Age	Age of onset	EDSS at time of examination
			Mean ± SD	Mean ± SD	Mean ± SD
MS patients	Male	49 (27%)	45.4 ± 7.7	29.1 ± 7.6	2.3 ± 1.7
	Female	131 (73%)	44.6 ± 8.2	29.5 ± 7.9	2.7 ± 1.1
	Overall	180	44.8 ± 8.0	29.4 ± 7.8	2.6 ± 1.3
	GENDER	*n*		AGE	
				Mean ± SD	

Healthy controls	Male	61 (29%)		45 ± 12	
	Female	152 (71%)		43 ± 11	
	Overall	213		44 ± 11.4	

MS: multiple sclerosis; EDSS: expanded disability status scale; SD: standard deviation. *P* < .05 indicates statistical significance.

**Table 2:** Association between CCR5Δ32 allele and EDSS scores and between CCR5Δ32 allele and age onset.

EDSS at time of examination				Genotype Δ32/+ (sex as covariate)				Genotype Δ32/+ (age as covariate)				Genotype Δ32/+ (age of onset as covariate)					Genotype Δ32/+	
	Mean ± SD	*t*	*P-*value	EDSS	*F*	*df*	*P*-value	EDSS	*F*	*df*	*P*-value	EDSS	*F*	*df*	*P*-value	Age of onset	*t*	*P*-value
WT	2.74 ± 1.31	2.04	.04		4.54	1.114	.03		5.12	1.114	.02		5.25	1.114	.02		(−)0.27	.78
Δ32/+	2.00 ± 1.26																	

Statistical analysis for association between Δ32 allele and EDSS scores was tested by Student's *t*-test and ANOVA methods.

MS: multiple sclerosis; EDSS: expanded disability status scale; *P* < .05 indicates statistical significance.

Statistical analysis for association between CCR5Δ32 allele and age onset was tested by Student's *t*-test. Age of onset was defined by the age at first manifestation of disease; *P* < .05 indicates statistical significance.

**Table 3:** Overview of major association studies.

Patients country	Clinical variable	Statistical method	Results	References
Iran	MS development	ANOVA	Yes (predisposing factor)	[29]
Brazil	Age of onset; EDSS	ANOVA; chi-square test; Mann-Whitney *U*-test	Potential favorable prognostic biomarker	[25]
Denmark	EDSS	Mann-Whitney *U*-test	No associations	[30]
Spain	Etiopathogeny of the disease	Not available	Yes (protective role)	[31]
Croatia; Slovenia	Age of disease onset or progression of the disease	Not available	No associations	[23]
USA	Age of onset; EDSS	Student's *t*-test; ANOVA	No associations	[22]
Northern Ireland	Age of onset; susceptibility to develop MS	Not available	No associations	[24]
Finland	Susceptibility to develop MS	chi-square test; ANOVA	No associations	[32]
Israel	EDSS	Not available	Potential favorable prognostic biomarker	[20]
Germany	Susceptibility to develop MS	ANOVA	No associations	[33]
USA	Age of onset	Not available	Age of onset was approximately 3 years later in patients carrying the CCR5Δ32 deletion	[19]
Denmark	Susceptibility to develop MS	ANOVA; Student's *t*-test	Lower risk	[21]
